# Alcohol in Patent Medicines

**Published:** 1891-10

**Authors:** 


### ALCOHOL IN PATENT MEDICINES.

A drink of whiskey is resorted to by the toper to "make him feel better." Alcohol seems to produce a temporary elation which many makers of patent medicines take advantage of. If a dose of the medicine seems to affect the patient at once, the presumption is that he will go on taking the remedy. Besides this, if the alcohol habit is once formed it will be hard work to discontinue taking the medicine, just as it is hard to stop drinking whiskey when the habit is once formed. Look out for a majority of the "bitters"—they are simply disguised alcohol.

In the report on nostrums, proprietary medicines and new drugs, which was read before the American Association for the cure of Inebriates, the analyses of a large number of well known patent medicines, disclosed alcohol in varying proportion.

We have grave doubts of the value of remedies of this nature It is on this account that after thoroughly testing its merits, we have endorsed the merits of HERBA VITA, advertised in this journal, as simple, harmless and efficacious. It is a real sin to dose the young with alcoholic mixtures.

